# Supplementary material for: Single-cell sequencing shows cellular heterogeneity of cutaneous lesions in lupus erythematosus
Source: Nat Commun. 2022 Dec 5;13:7489. doi: 10.1038/s41467-022-35209-1 (PMC9722937; doi:10.1038/s41467-022-35209-1)
Supplement: Supplementary file 1 — Supplementary Information [file 41467_2022_35209_MOESM1_ESM.pdf]

Supplementary Information

Single-cell sequencing shows cellular heterogeneity of cutaneous lesions in lupus erythematosus

Meiling Zheng[1,2†], Zhi Hu[1,2†], Xiaole Mei[3†], Lianlian Ouyang[1,2†], Yang Song[1,2†], Wenhui Zhou[1,2], Yi Kong[1,2], Ruifang Wu[1,2], Shijia Rao[1,2], Hai Long[1,2], Wei Shi[4], Hui Jing[1,2], Shuang Lu[1,2], Haijing Wu[1,2], Sujie Jia[5], Qianjin Lu[1,2,3*], Ming Zhao[1,2*]

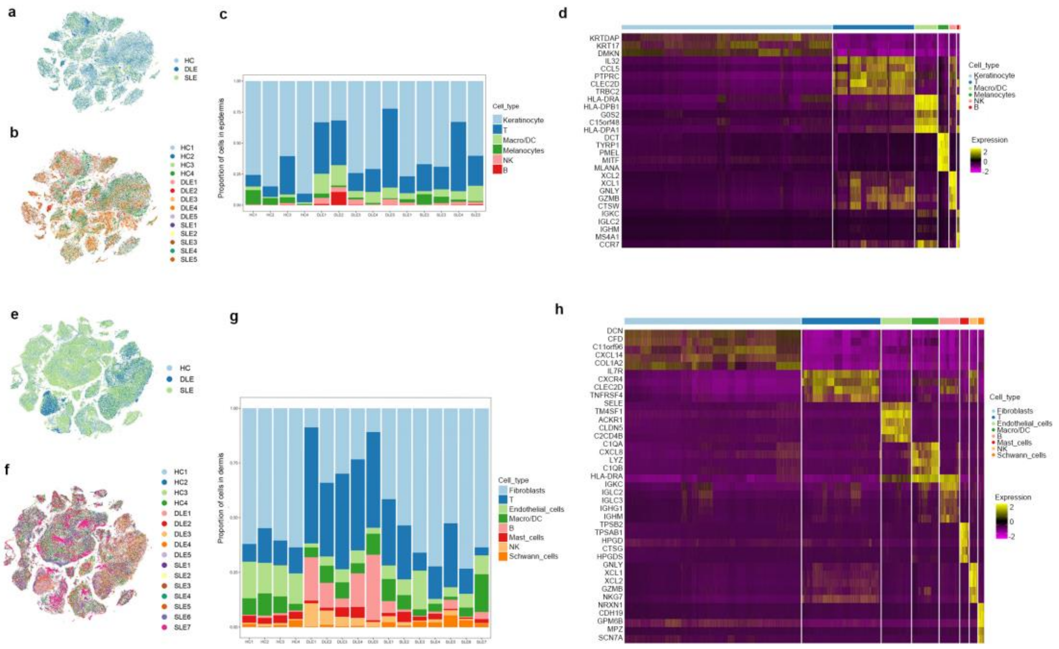

Supplementary Figure 1. scRNA-seq shows altered cell composition of cutaneous lesions in patients with DLE and SLE. a, e. t-SNE plots show the distribution of cells from HC, DLE and SLE in epidermis(a) or dermis(e). **b, f.** t-SNE plot show the distribution of individual samples in epidermis(b) or dermis(f). **c, g.** Bar plots show the epidermal(c) or dermal(g) cell type proportions in each sample. Macro/DC: macrophages/dendritic cell. **d.h.** Heatmap represent gene expression of the selected genes expression for 6 epidermal cell types(d) or 8 dermal cell types(h). The color of column annotation represents different cell type. The high expression was represented by the color of yellow. Source data are provided as a Source Data file.

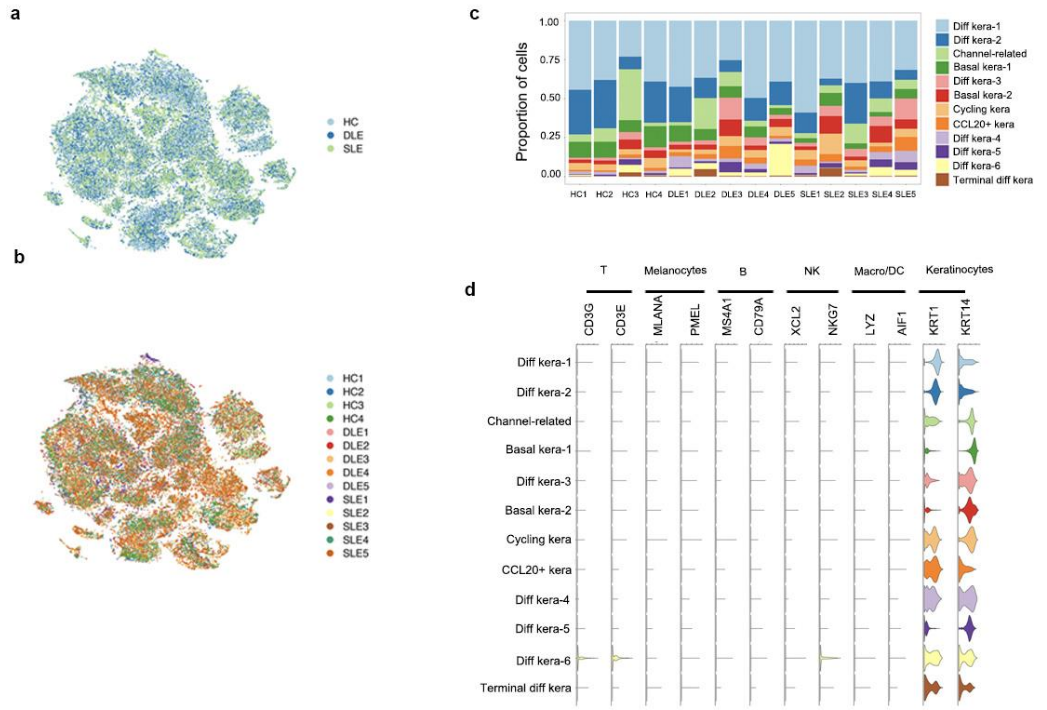

Supplementary Figure 2. scRNA-seq shows keratinocyte subtypes in DLE and SLE. a, b. t-SNE plots show sample group (a) and individuals (b) distribution in the second sub-clustering analysis of keratinocytes. **c.** A bar plot represents the proportions of keratinocyte subtypes in each healthy control and patients with lupus erythematosus (LE). Diff kera-1-6: differentiating keratinocyte subtype 1-6; Basal kera-1,2: basal keratinocyte subtype 1,2; Channel-related kera: channel-related keratinocyte; Cycling kera: cycling keratinocyte; CCL20+ kera: CCL20+ keratinocyte subtype; Terminal diff kera: terminally differentiating keratinocyte subtype. **d.** A stacked violin plot shows the classical cell type gene expression in labeled 12 keratinocyte subtypes. Source data are provided as a Source Data file.

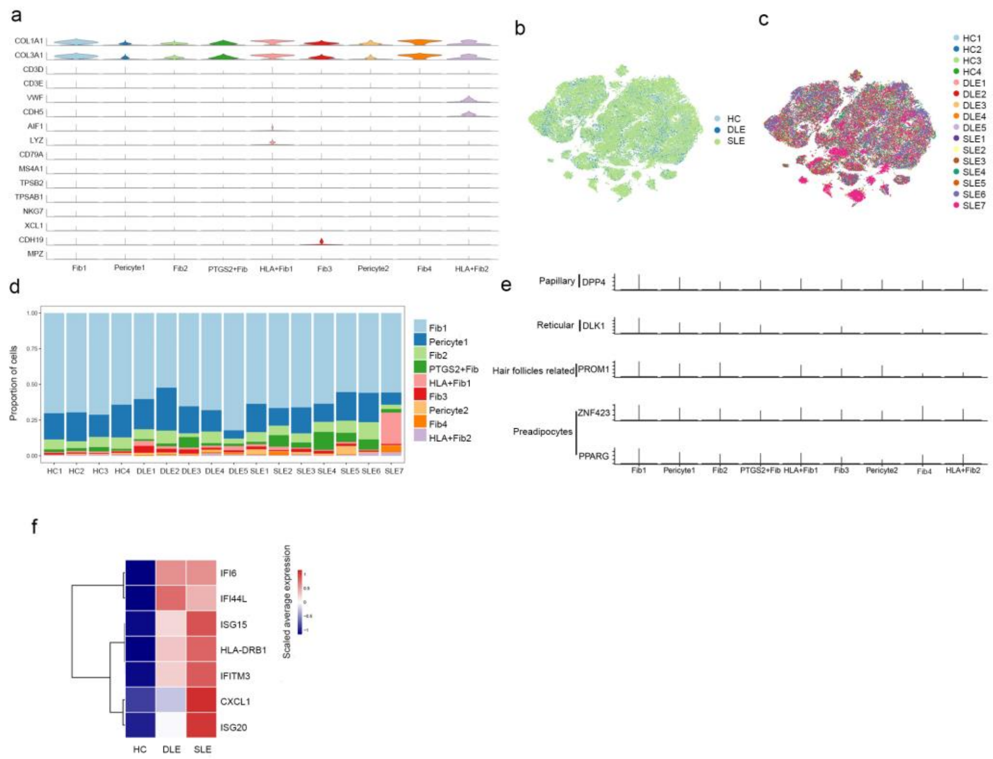

Supplementary Figure 3. scRNA-seq shows CXCL1^{+} fibroblasts and other amplified fibroblast subtypes in DLE and SLE. a. Stacked violin plot shows main dermal cell type marker genes expression in identified 9 fibroblast subtypes. Fib1-4: Fibroblast subtype 1-4; HLA^{+}Fib: HLA^{+} Fibroblast; CXCL1^{+}Fib: CXCL1^{+}Fibroblast. **b, c.** t-SNE plots show the sample group (b) and individuals (c) distribution after second sub-clustering analysis of fibroblasts. **d.** Bar plots show the proportions of 9 fibroblast subtypes in each sample of HC, DLE and SLE. **e.** Stacked violin plot shows classical fibroblast subtype genes expression in 9 fibroblast subtypes. **f.** A heatmap shows selected genes expression in fibroblasts of HC, DLE and SLE. Source data are provided as a Source Data file.

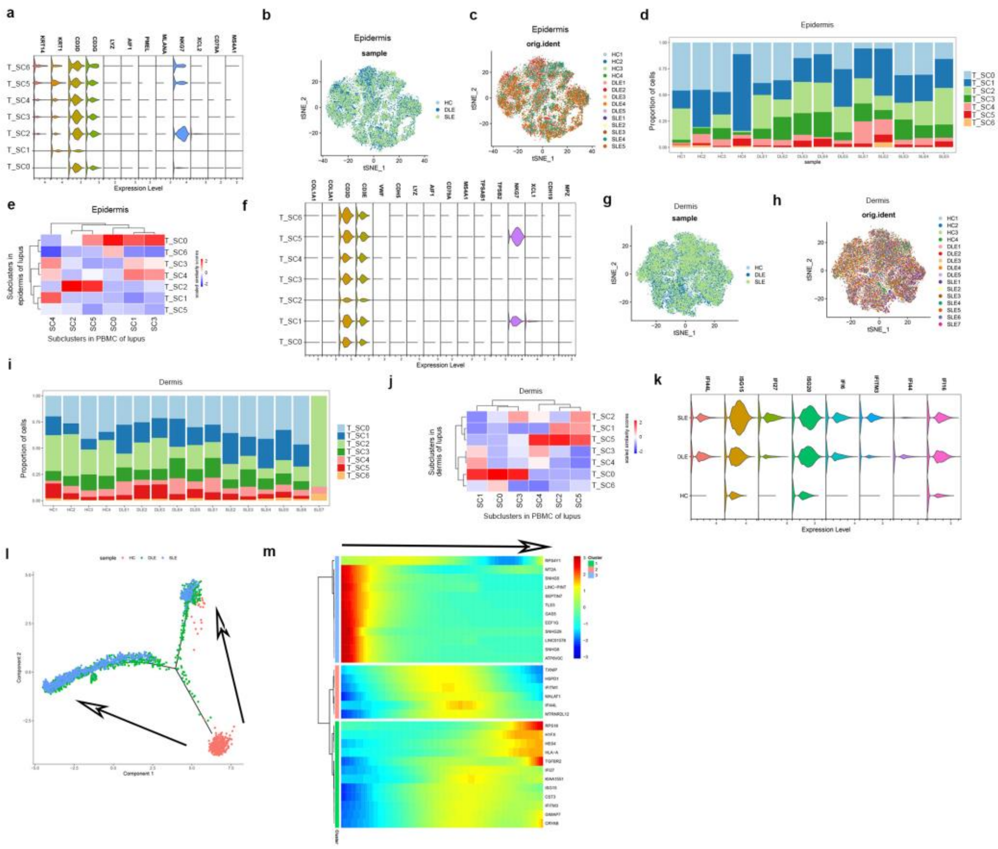

Supplementary Figure 4. The distribution of T cell subclusters in skin and similarity scores of T cell SCs between PBMC and skin tissues. a, f. Stack violin plots show major epidermal and dermal cell type marker genes expression in epidermal(a) or dermal(f) T cell subclusters(T_SC). **b, c, g, h.** t-SNE plots show the distributions of sample group(b, g) and individual samples(c, h) in epidermal(b, c) and dermal(g, h) T cell sub-clustering analysis. **d, i.** Bar plots represent the proportions of epidermal(d) and dermal(i) T cell SCs in each individual samples. **e, j.** Heatmaps show similarity score of epidermal(e) or dermal(j) T cell SCs comparing to T_SCs in PBMC data of lupus reported by Nehar-Belaid, D. The high similarity scores were identified by the red color. **k.** A stack violin plot shows ISGs expression in dermal T cells of HC, DLE and SLE. **l.** Trajectory plot shows pseudotime trajectory analysis of dermal T cells from HC, DLE and SLE by Monocle2, colors represent different sample group. The

arrow indicates the possible differentiation direction. **m.** A heatmaps shows a series of gene expressions in the differentiation state which were predicted by pseudotime trajectory analysis. The arrow indicates the possible differentiation direction. Source data are provided as a Source Data file.

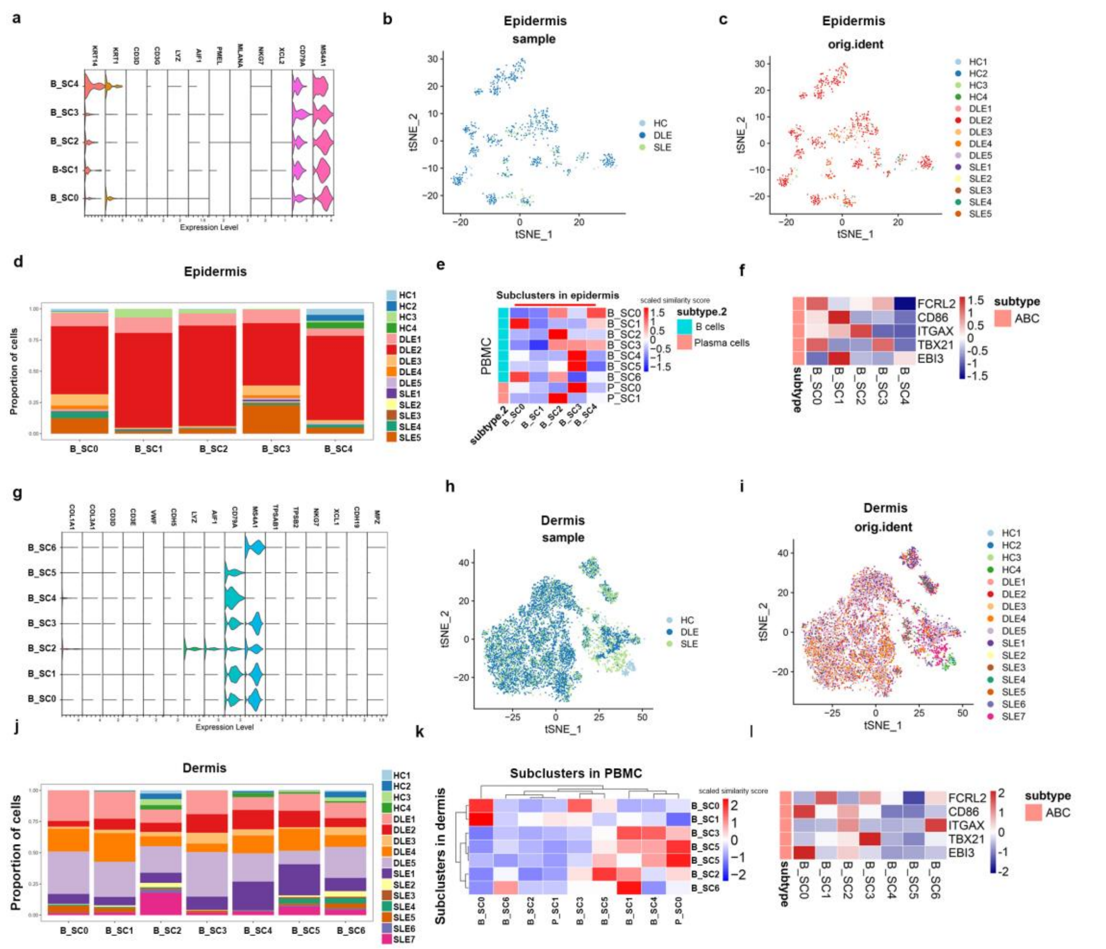

Supplementary Figure 5. The distribution of B cell subclusters in skin and similarity scores of B cell SCs between PBMC and skin tissues. a, g. Stack violin plots show major epidermal and dermal cell type marker genes expression in epidermal(a) or dermal(f) B cell subclusters. **b, c, h, i.** t-SNE plots show the distributions of sample group(b, h) and individual samples(c, i) in epidermal(b, c) and dermal(h, i) B cell sub-clustering analysis. **d, j.** Bar plots represent the proportions of cells from HC, DLE and SLE in epidermal(d) or dermal(j) B cell subclusters. **e, k.** Heatmaps show similarity score of epidermal(e) or dermal(k) B cell SCs between PBMC data of lupus reported by Nehar-Belaid, D. The high scores were identified by red color. **f, l.** Heatmaps show classical marker genes of age associated B cells (ABC)

in epidermal(f) or dermal B(l) cell subclusters. Source data are provided as a Source Data file.

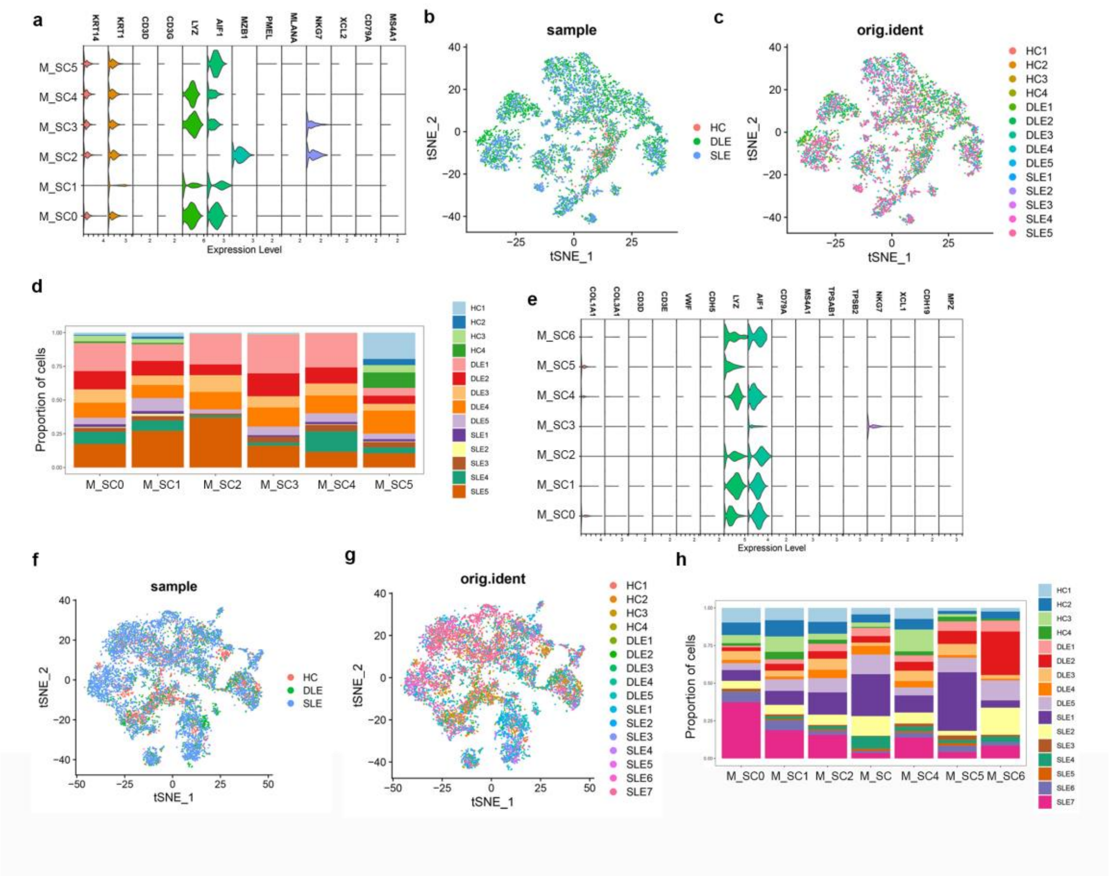

Supplementary Figure 6. The distributions of Macro/DC subclusters in each individual sample. a, e. Stack violin plots show major epidermal and dermal cell type marker genes expression in epidermal(a) or dermal(f) Macro/DC subclusters. **b, c, f, g.** t-SNE plots show the distributions of sample group(b, f) and individual samples(c, g) in epidermal(b, c) and dermal(f, g) Macro/DC sub-clustering analysis. **d, h.** Bar plots represent the proportions of cells from HC, DLE and SLE in epidermal(d) or dermal(i) Macro/DC subclusters. Source data are provided as a Source Data file.

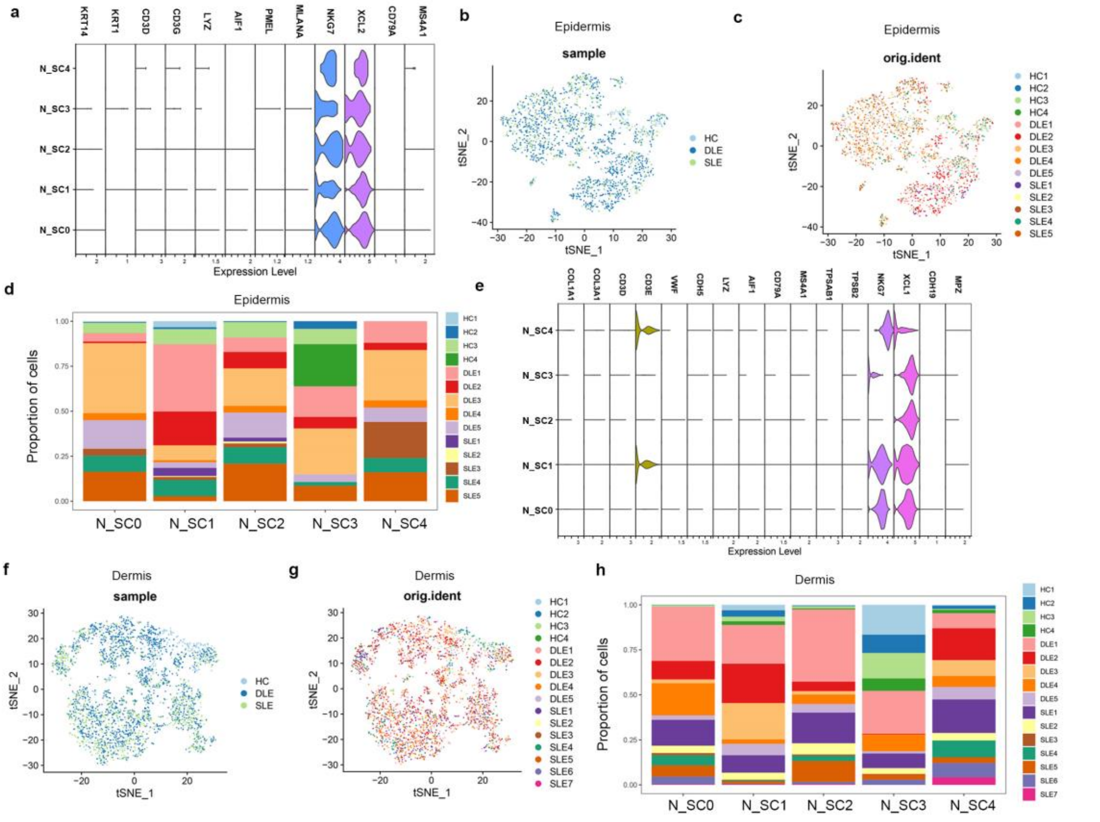

Supplementary Figure 7. Distribution of NK cell subclusters in individual sample. **a, e.** Stack violin plots show major epidermal and dermal cell type marker genes expression in epidermal(a) or dermal(f) NK subclusters. **b, c, f, g.** t-SNE plots show the distributions of sample group(b, f) and individual samples(c, g) in epidermal(b, c) and dermal(f, g) NK sub-clustering analysis. **d, h.** Bar plots represent the proportions of cells from HC, DLE and SLE in epidermal(d) or dermal(i) NK subclusters. Source data are provided as a Source Data file.

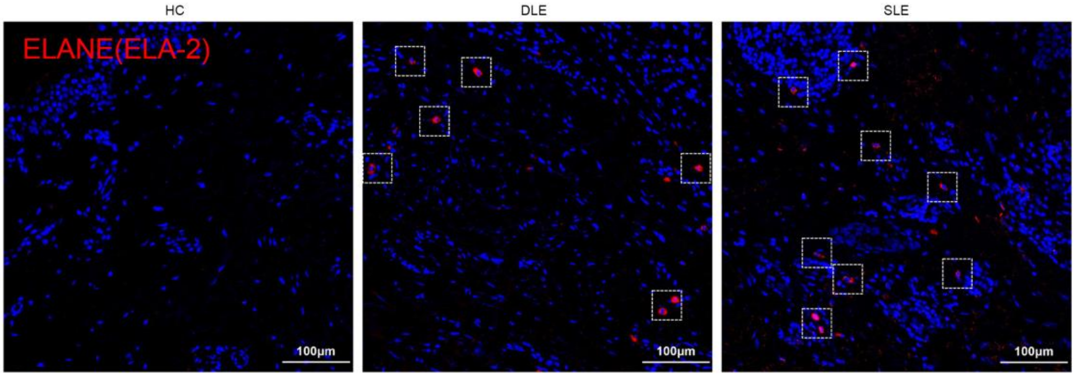

Supplementary Figure 8. Neutrophil infiltration in cutaneous lesion of DLE and SLE. Representative confocal images of immunofluorescence staining for ELANE (neutrophils, red). Scale bar: 100μM. Data are representative of 3 independent experiments. Source data are provided as a Source Data file.
