# Supplementary material for: Single-cell sequencing shows cellular heterogeneity of cutaneous lesions in lupus erythematosus
Source: Nat Commun. 2022 Dec 5;13:7489. doi: 10.1038/s41467-022-35209-1 (PMC9722937; doi:10.1038/s41467-022-35209-1)
Supplement: Supplementary file 3 — Description of Additional Supplementary Files [file 41467_2022_35209_MOESM3_ESM.pdf]

Description of Additional Supplementary Files

Supplementary Data 1: Personal information for each sample included in this study (5 healthy controls, 8 DLE patients and 10 SLE patients)

Supplementary Data 2: Single cell sequencing information for individual sample included in the study (14 epidermis and 16 dermis)

Supplementary Data 3: Differentially expressed genes (DEGs) in each cell type of the epidermis dataset.

Supplementary Data 4: Differentially expressed genes (DEGs) in each cell type of the dermis dataset.

Supplementary Data 5: Differentially Expressed Genes (DEGs) in each subtype of keratinocytes dataset with the cutoff by logFC≥0.25, P value＜0.05.

Supplementary Data 6: GO BP terms enriched in expanded keratinocyte subtypes.

Supplementary Data 7: Differentially Expressed Genes (DEGs) in each subtype of fibroblasts dataset with the cutoff by logFC≥0.25, P value＜0.05.

Supplementary Data 8: GO BP terms enriched in expanded fibroblast subtypes.

Supplementary Data 9: Differentially Expressed Genes (DEGs) in each subcluster (epidermis: n=7, T_SC0-T_SC6; dermis: n=7, T_SC0-T_SC6) of T cells in the epidermis and dermis dataset with the cutoff by logFC≥0.25, P value＜0.05.

Supplementary Data 10: Differentially Expressed Genes (DEGs) in each subcluster (epidermis: n=5, B_SC0-B_SC4; dermis: n=7, B_ SC0-B_SC6) of B cells in the epidermis and dermis dataset with the cutoff by logFC≥0.25, P value＜0.05.

Supplementary Data 11: Differentially Expressed Genes (DEGs) in each subcluster (epidermis: n=6, SC0-SC5; dermis: n=7, SC0-SC6) of Mac/DCs in the epidermis and dermis dataset with the cutoff by logFC≥0.25, P value＜0.05.

Supplementary Data 12: Differentially Expressed Genes (DEGs) in each subcluster (epidermis: n=5, SC0-SC4; dermis: n=5, SC0-SC3) of NK cells in the epidermis and dermis dataset with the cutoff by logFC≥0.25, P value＜0.05.

Supplementary Data 13: Ligand-Receptors expressed in epidermal cells of HCs, DLE and SLE with the cutoff by P value＜0.05.

Supplementary Data 14: Ligand-Receptors expressed in dermal cells of HCs, DLE and SLE with the cutoff by P value＜0.05.
