# Supplementary material for: Single-cell sequencing shows cellular heterogeneity of cutaneous lesions in lupus erythematosus
Source: Nat Commun. 2022 Dec 5;13:7489. doi: 10.1038/s41467-022-35209-1 (PMC9722937; doi:10.1038/s41467-022-35209-1)
Supplement: Supplementary file 18 — Reporting Summary [file 41467_2022_35209_MOESM18_ESM.pdf]

nature portfolio

Corresponding author(s): Ming Zhao; Qianjin Lu

Last updated by author(s): Oct 17, 2022

Reporting Summary

Nature Portfolio wishes to improve the reproducibility of the work that we publish. This form provides structure for consistency and transparency in reporting. For further information on Nature Portfolio policies, see our Editorial Policies and the Editorial Policy Checklist.

Statistics

For all statistical analyses, confirm that the following items are present in the figure legend, table legend, main text, or Methods section.

n/a	Confirmed	
☐	☒	The exact sample size (*n*) for each experimental group/condition, given as a discrete number and unit of measurement
☐	☒	A statement on whether measurements were taken from distinct samples or whether the same sample was measured repeatedly
☐	☒	The statistical test(s) used AND whether they are one- or two-sided<br>*Only common tests should be described solely by name; describe more complex techniques in the Methods section.*
☒	☐	A description of all covariates tested
☐	☒	A description of any assumptions or corrections, such as tests of normality and adjustment for multiple comparisons
☐	☒	A full description of the statistical parameters including central tendency (e.g. means) or other basic estimates (e.g. regression coefficient) AND variation (e.g. standard deviation) or associated estimates of uncertainty (e.g. confidence intervals)
☒	☐	For null hypothesis testing, the test statistic (e.g. *F*, *t*, *r*) with confidence intervals, effect sizes, degrees of freedom and *P* value noted<br>*Give P values as exact values whenever suitable.*
☒	☐	For Bayesian analysis, information on the choice of priors and Markov chain Monte Carlo settings
☒	☐	For hierarchical and complex designs, identification of the appropriate level for tests and full reporting of outcomes
☒	☐	Estimates of effect sizes (e.g. Cohen's *d*, Pearson's *r*), indicating how they were calculated

Our web collection on statistics for biologists contains articles on many of the points above.

Software and code

Policy information about availability of computer code

Data collection	No special or proprietary software was used.
Data analysis	The following commercial software were used in this study:<br>Raw sequencing data were first filtered and then mapped to GRCh38 by using Cell Ranger Version 3.0.0. Cell doublets were removed by Scrublet(vO.l) Then, the Seurat (v4.0),Harmony(v1.0), Monocle (v2.0.0) ,pheatmap(v1.0.12),ggplot2(v3.3.5),dplyr(v1.0.7),RColorBrewer(v1.1-2),clusterProfiler(v3.18.1),were used as the main tool for single-cell sequencing analysis in R(4.0.3). CellPhoneDB(v2.0) was used for cell-cell communication analysis. The inForm software(v2.4.2) was used for the image analysis of immunofluorescence staining. Details are described in Methods.<br>The code for analysing data in this study was presented in GitHub (https://doi.org/10.5281/zenodo.7193545).

For manuscripts utilizing custom algorithms or software that are central to the research but not yet described in published literature, software must be made available to editors and reviewers. We strongly encourage code deposition in a community repository (e.g. GitHub). See the Nature Portfolio guidelines for submitting code & software for further information.

Data

Policy information about availability of data

All manuscripts must include a data availability statement. This statement should provide the following information, where applicable:

- Accession codes, unique identifiers, or web links for publicly available datasets
- A description of any restrictions on data availability
- For clinical datasets or third party data, please ensure that the statement adheres to our policy

The data of this single cell RNA sequencing is available in the GEO database with the accessible codes of GSE179633. The code of our program was present in GitHub (https://github.com/zml314/skin).
The Human reference (GRCh38) dataset required for Cell Ranger is avaliable at https://cf.10xgenomics.com/supp/cell-exp/refdata-gex-GRCh38-2020-A.tar.gz.

Human research participants

Policy information about studies involving human research participants and Sex and Gender in Research.

Reporting on sex and gender	We provided messages of gender in supplementary table S1. Totally, we collected 5 skin tissues from male patients and 18 skin tissues from female patients.
Population characteristics	We collected the age, sex and other clinical information of all samples in Table S1.
Recruitment	All skin biopsies were collected at the dermatology biopsy center in the Second Xiangya Hospital of Central South University, Xiangya Hospital of Central South and University and Institute of Dermatology of Chinese Academy of Medical Sciences and Peking Union Medical College.
Ethics oversight	Our study was approved by the ethics committee of the Second Xiangya Hospital,Xiangya Hospital of Central South and Institute of Dermatology of Chinese Academy of Medical Sciences and Peking Union Medical College.

Note that full information on the approval of the study protocol must also be provided in the manuscript.

Field-specific reporting

Please select the one below that is the best fit for your research. If you are not sure, read the appropriate sections before making your selection.

☒ Life sciences ☐ Behavioural & social sciences ☐ Ecological, evolutionary & environmental sciences

For a reference copy of the document with all sections, see nature.com/documents/nr-reporting-summary-flat.pdf

Life sciences study design

All studies must disclose on these points even when the disclosure is negative.

Sample size	No statistical method was used to predetermine sample size.
Data exclusions	We removed low-quality cells whose nFeature_RNA was less than 200 or greater than 5000, percent.mt was more than 20% and percent.redcell was more than 10%. Due to sequencing bias, there were some other cells involved in the second clustering analysis of a cell type. To eliminate their interference, we removed these cells before sub-clustering analysis.
Replication	All experiments of immunofluorescence staining in our study replicated 3 times.All replications were successful,and the details are provided in corresponding figure legend.
Randomization	All skin biopsy tissue samples for single-cell RNA sequence and immunofluorescence staining were collected randomly in this study.
Blinding	Blinding is not applicable in this study for the reason that there was no specific grouping and intervention.

Reporting for specific materials, systems and methods

We require information from authors about some types of materials, experimental systems and methods used in many studies. Here, indicate whether each material, system or method listed is relevant to your study. If you are not sure if a list item applies to your research, read the appropriate section before selecting a response.

Materials & experimental systems

n/a	Involved in the study
☐	☒ Antibodies
☒	☐ Eukaryotic cell lines
☒	☐ Palaeontology and archaeology
☒	☐ Animals and other organisms
☒	☐ Clinical data
☒	☐ Dual use research of concern

Methods

n/a	Involved in the study
☒	☐ ChIP-seq
☒	☐ Flow cytometry
☒	☐ MRI-based neuroimaging

Antibodies

Antibodies used	Antibody (Supplier name, dilutions, catalog number, clone name)<br>CD3 (MXB Biotech, without dilutions, MAB-0740, MX036)<br>CD19 (Abcam, 1:1000, ab134114,EPR5906)<br>CD56 (Abcam, 1:400, ab75813, EP2567Y)<br>CCL20 (Abcam, 1:50, ab224188, EPR22376-58)<br>KRT10 (Abcam, 1:500, ab76318, EP1607IHCY)<br>CXCL1 (Abcam, 1:100, ab89318, MM0208-9A18)<br>HLA-DRB1 (Abcam, 1:1000, ab133578, EPR6148)<br>Vimentin (Abcam, 1:20000, ab92547, EPR3776)<br>ELANE (Abcam, 1:2000, ab131260, EPR7479)<br>CCL19 (ProteinTech, 1:500, 13397-1-AP, AG4200)<br>CCR7 (ProteinTech, 1:500, 25898-1-AP, AG22941)<br>CD68(Abcam, 1:400, ab955, KPI).<br><br>The second antibodies used in this study ：Donkey Anti-Rabbit IgG, Abcam, 1:1000, ab205722, HRP.
Validation	All antibodies are commercially available and their manufacturers provided their validation documents. They were validated for IHC.<br>http://www.maxim.com.cn/sitecn/dklkthdklkt/7424.html<br>https://www.abcam.cn/cd19-antibody-epr5906-ab134114.html<br>https://www.abcam.cn/cd68-antibody-kp1-ab955.html<br>https://www.abcam.cn/ncam1-antibody-ep2567y-ab75813.html<br>https://www.abcam.cn/macrophage-inflammatory-protein-3-alpha-antibody-epr22376-58-ab224188.html<br>https://www.abcam.cn/cytokeratin-10-antibody-ep1607ihcy-cytoskeleton-marker-ab76318.html<br>https://www.abcam.com/hla-class-ii-drb1-antibody-epr6148-ab133578.html<br>https://www.abcam.cn/cxcl1gro-alpha-antibody-mm0208-9a18-ab89318.html<br>https://www.abcam.cn/vimentin-antibody-epr3776-cytoskeleton-marker-ab92547.html<br>https://www.abcam.cn/neutrophil-elastase-antibody-epr7479-ab131260.html<br>https://www.ptgcn.com/products/CCR7-Antibody-25898-1-AP.html<br>https://www.ptgcn.com/products/CCL19-Antibody-13397-AP.html
